# Comparison and Agreement Between Traditional and Smartphone-Camera-Based Morphometric Measurements in Holstein and Simmental Cattle

**DOI:** 10.3390/vetsci13050502

**Published:** 2026-05-21

**Authors:** Yavuzkan Paksoy, İbrahim Erez, Muhammet Hanifi Selvi

**Affiliations:** 1 Department of Animal Science and Animal Nutrition, Division of Animal Husbandry, Ceyhan Faculty of Veterinary Medicine, Cukurova University, Adana 01950, Turkey; 2 Center for Experimental Research and Application in Health Sciences, Cukurova University, Adana 01950, Turkey; ierez@cu.edu.tr; 3 Department of Animal Science and Animal Nutrition, Faculty of Veterinary Medicine, Necmettin Erbakan University, Konya 42310, Turkey; mselvi@erbakan.edu.tr

**Keywords:** cattle, morphometric measurement, image analysis, animal welfare, precision livestock farming

## Abstract

Accurate determination of morphometric body measurements is essential for monitoring growth, evaluating production traits, and supporting selection decisions in cattle breeding. However, traditional measurement methods require direct contact with animals, which may increase labor requirements, negatively affect animal welfare, and pose safety risks for operators. This study evaluated the relationship and agreement between traditional tape measurements and smartphone-camera-based morphometric measurements in cattle. A total of 100 cattle raised in the Mediterranean region of Türkiye, including 50 Holstein and 50 Simmental animals, were included in the study. Withers height, body length, rump height, and forechest width were measured using both conventional tools and a smartphone-camera-based method. Regression analyses demonstrated strong linear relationships between methods, particularly for body length and withers height (R^2^ = 0.564–0.961). Bland–Altman analysis revealed small but significant systematic differences between methods, with camera-based measurements generally producing slightly higher values than tape measurements. The strongest agreement was observed for body length measurements, whereas wider limits of agreement were detected for anatomically complex traits, such as rump height and forechest width. Although the findings support the potential applicability of smartphone-based morphometric measurements as a practical and contactless alternative under field conditions, measurements were obtained only from a single lateral view, which should be considered an important methodological limitation. Future studies using multi-view or three-dimensional imaging systems may further improve measurement accuracy and agreement.

## Simple Summary

Accurate determination of morphometric body measurements is essential for monitoring growth, evaluating production traits, and supporting selection decisions in cattle breeding. However, traditional measurement methods require direct contact with animals, which may increase labor requirements, negatively affect animal welfare, and pose safety risks for operators. This study aimed to evaluate the agreement between traditional measurement methods and an image-based measurement approach using a smartphone camera for determining key morphometric traits in cattle. Withers height, body length, rump height, and forechest width were measured using both conventional tools and a camera-based method. The findings suggest that image-based measurement methods can serve as a practical and contactless alternative in cattle production systems, offering advantages in terms of animal welfare and operator safety.

## 1. Introduction

To achieve sustainable livestock farming, strategies that better integrate practices, such as animal welfare, genetic and environmental improvement, animal health, and marketing, into herd management must be developed. Morphometric measurements are important for determining breed standards, identifying deficiencies in care and feeding opportunities in herd management, and assessing growth and development capabilities. As animals grow, their body size and live weight tend to increase. This increase continues steadily in healthy animals until they reach maturity. In animal husbandry, the growth and development of animals are monitored through regular measurements and weighings [1,2]. Certain body measurements taken from animals provide information about their physical condition in herd management, and it is known that there is a high correlation between the data obtained and live weights [3,4,5]. In dairy cow breeding programs, body weight and body condition score are critically important. These two parameters are crucial for achieving optimum yield and monitoring animal health. However, problems can arise due to the lack of scales in most commercial farms and the difficulty in always accessing experienced veterinarians who can determine body condition scores [2,6]. Automatic animal health surveillance systems have become increasingly popular recently [7,8].

The live weights of animals can generally be accurately determined in facilities using scales. In addition to weighing, simple and easy measurements taken from different parts of the animals using a measuring stick or measuring tape can be used as an alternative method for estimating live weight. Body measurements vary according to important factors such as sex, type of yield, age, and breed [9].

In cattle breeding, in addition to keeping regular records, careful evaluation of phenotypic traits provides valuable information about the animal’s health status and productivity potential [10]. The heritability levels of various body measurements, such as withers height, rump height, body length, forechest width and circumference, and shank circumference, are moderate to high [6,8,9]. Nowadays, in all countries where breeding programs are implemented, conformation traits are used as important selection criteria. Methods such as measurement and weighing are frequently preferred [11,12].

Cattle are typically weighed using scales with a capacity of one ton, and systems enclosed by fencing are preferred to ensure the animal can be weighed comfortably and safely. It is crucial that the animal remains calm during measurement and maintains its natural stance on a level surface. The accuracy of the measurements is increased if the personnel taking the measurements treat the animals gently and act in a way that calms particularly skittish and fearful animals. Because if animals are not treated properly, it is impossible to keep them still [13].

Europe was the origin of the Holstein–Friesian breed; then, European Holstein–Friesian cattle were brought to North America, where they were subjected to separate selection. From there, these cattle returned to Europe, already of the US Holstein type, but they also spread worldwide. This breed is the most widely bred in the world. It has typical dairy characteristics, with a body that gradually widens from front to back. It should be noted that primiparous Holstein cows may continue to grow after first calving, which can influence morphometric measurements [14,15]. Their withers height is 145–156 cm. The Simmental breed originates from Switzerland, Germany, and Austria. It is oriented towards combined (meat–milk) yield. It is also preferred due to its adaptation to the climate and environmental conditions of our country [12,14,16,17].

Important body measurements in cattle breeds, such as withers height, are determined by a combination of genetic factors, nutritional status, and environmental factors. The genetic makeup of parents influences the anatomical structure of the offspring. In addition, the care and feeding of the offspring after birth have an impact on growth and development. These measurements have significant effects on cattle health and performance [5,9,12]. The replacement of traditional measurement methods with technological tools such as smartphones and cameras not only improves animal welfare but also protects people from the risk of injury [13]. Recent studies report that body measurements in animals can be measured more easily and effectively using image analysis [18,19,20]. In this study, measurements obtained using traditional methods and digital methods using smartphones were compared in terms of consistency. Therefore, body measurements frequently taken from cattle of various breeds and different genders were obtained using both digital and traditional methods, and the degree of agreement between the two methods was evaluated. Despite the increasing use of image-based techniques in livestock research, there is still a need to evaluate their accuracy and agreement with traditional measurement methods under field conditions. Therefore, the hypothesis of this study is that morphometric measurements obtained using a smartphone-based imaging method show strong agreement and predictive capability compared to traditional tape measurements. In addition, this study aims to assess whether this agreement is consistent across different cattle breeds with distinct morphological characteristics.

## 2. Materials and Methods

This study was approved by the Çukurova University Local Ethics Committee for Animal Experiments (Sağlık Bilimleri Deneysel Uygulama ve Araştırma Merkezi—Health Sciences Experimental Application and Research Center) under decision number 9, dated 23 December 2025. All procedures involving animals were conducted in accordance with institutional and national guidelines for animal welfare and ethical use of animals in research.

### 2.1. Study Area and Animal Material

This study was conducted in the Mediterranean region of Türkiye. The Mediterranean region, located on the southern coast of Türkiye, is known for its typical Mediterranean climate with hot, dry summers and mild, rainy winters, as well as its maquis vegetation and rugged topography where mountains run parallel to the coast. A total of 100 cattle (50 Holstein and 50 Simmental) of different ages (1.5–3 years) raised on private farms were included in the study. To ensure the accuracy of the analyses, similar age and gender groups were used, and furthermore, a power test was conducted to ensure the appropriateness of the numbers. Animals were randomly selected, and no anesthetic agents were used during measurements. Animals that could not be safely measured were excluded from the study. Information on age, sex, and management practices was obtained from farm records. Animals were selected based on availability and handling feasibility from private farms in the study region. Inclusion criteria required animals to be clinically healthy (determined with the help of the farm’s veterinarian), standing unassisted, and safely accessible for both manual and image-based measurements. Information regarding age, sex, and physiological status (e.g., lactating or non-lactating) was obtained from farm records and considered during data interpretation.

### 2.2. Housing and Feeding

Animals were reared under routine farm management conditions. Diets consisted of roughage (hay or pasture) and concentrate feeds formulated according to the animals’ growth stage and production status. Although detailed feed intake and daily weight gain were not individually recorded, feeding practices were consistent with standard regional livestock management systems [21,22].

### 2.3. Morphometric Measurements

All measurements were taken while animals were standing naturally on a flat surface. Height measurements were performed using a measuring stick, while length and width measurements were taken using a flexible measuring tape [6,23]. All measurements, both traditional and image-based, were performed by the same trained operator to minimize inter-observer variability.

For the image-based method, photographs were obtained using an iPhone 16 (USA) smartphone camera (48 MP Fusion Main Camera: 26 mm, ƒ/1.6 aperture, sensor-based optical image stabilization, 100% Focus Pixels). All images were captured under standardized field conditions. The camera was positioned approximately 3 m from the animal and 1 m above ground level, with the optical axis maintained perpendicular to the animal’s body to minimize geometric distortion. Photographs were taken only from the left lateral side of the animals while standing stationary in a normal resting posture.

To reduce environmental variability, photographs were obtained during daylight under similar lighting conditions and without excessive shadowing. Four consecutive images were captured for each animal and morphometric trait. Among these images, the photograph showing the clearest visualization of anatomical landmarks and the most stable posture was selected for analysis. Images showing motion artifacts, excessive body rotation, limb displacement, or unclear anatomical boundaries were excluded.

**Figure 1:**
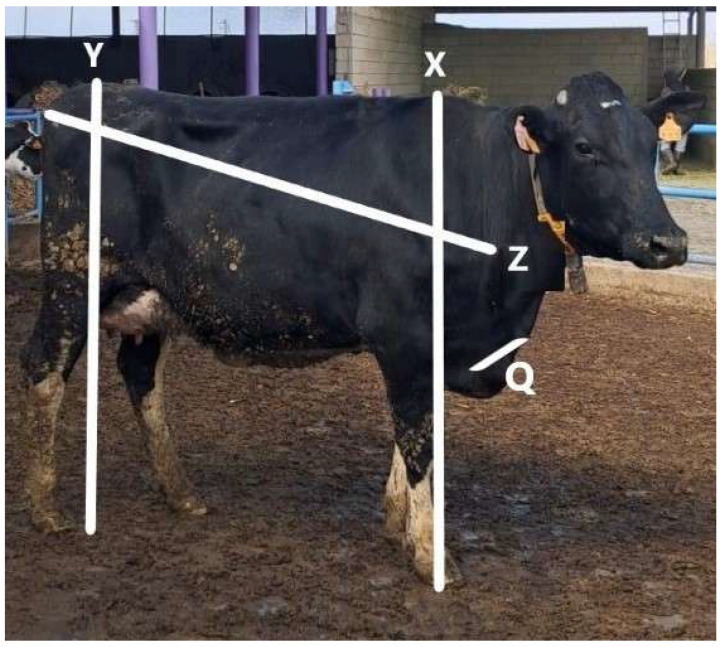
Anatomical landmarks and morphometric traits were evaluated using traditional and smartphone-camera-based measurements. X: withers height; Y: rump height; Z: body length; Q: forechest width.

No automated image-processing or artificial intelligence-based software was used during the measurement process. All image-based measurements were manually performed using a smartphone measurement application based on predefined anatomical landmarks. Anatomical reference points, including the withers, rump, shoulder point, and tuber ischii, were identified according to standard morphometric definitions reported in previous studies [3,5,9,13,24] (Figure 1).

All anatomical landmarks were manually identified by the same operator directly on the smartphone images, and no automated landmark detection or artificial intelligence-based image analysis system was used.

To improve consistency between animals, image acquisition conditions, including camera distance, camera height, and viewing angle, were standardized during all measurements (the Apple Measure app) [13]. Real-world dimensions were estimated using the smartphone measurement application based on proportional scaling principles under fixed-distance imaging conditions. Despite these precautions, slight deviations related to animal posture, hair coat thickness, respiratory movement, and operator-dependent landmark identification could not be completely eliminated.

The evaluated morphometric traits were as follows.

• Withers height: Vertical distance from the highest point of the withers to the ground.
• Rump height: Vertical distance from the highest point of the rump to the ground.
• Forechest width: Horizontal distance between the shoulder points.
• Body length: Distance between the point of the shoulder and the lateral angle of the tuber ischii [25].

### 2.4. Statistical Analysis

All statistical analyses were performed using IBM SPSS Statistics (Version 26.0; IBM, Armonk, NY, USA). Descriptive statistics (mean, standard deviation, minimum, and maximum values) were calculated for all morphometric traits. The normality of data distribution was assessed using the Shapiro–Wilk test. For comparisons between two measurement methods (traditional vs. image-based), paired sample *t*-tests were applied. When comparisons involved more than two groups (e.g., breed differences), one-way analysis of variance (ANOVA) was used, followed by post hoc tests (Tukey) when appropriate. Pearson correlation analysis was performed to evaluate the relationships between measurement methods. Linear regression analysis was conducted to assess the predictive relationship between image-based measurements and traditional measurements. In regression models, traditional measurements were considered as the dependent variable, while image-based measurements were treated as independent predictors. Regression analysis was specifically used to determine whether image-based measurements could reliably predict traditional measurements.

Agreement between the two measurement methods was further evaluated using the Bland–Altman method, which allows visualization of the mean difference (bias) and the 95% limits of agreement (mean difference ± 1.96 SD) between measurements.

In addition, simple linear regression analysis was conducted to determine the predictive capability of camera-based measurements for estimating tape-measured morphometric traits. The strength of the relationship between the two methods was evaluated using the coefficient of determination (R^2^).

All statistical tests were performed at a 95% confidence level, and differences were considered statistically significant at *p* < 0.05.

In addition to the coefficient of determination (R^2^), regression coefficients (intercept and slope) and their statistical significance (*p*-values) were reported to provide a more comprehensive evaluation of the predictive relationships between image-based and traditional measurements.

## 3. Results

As shown in Table 1, mean values of body measurements obtained by traditional and image-based methods were generally similar, although slight differences were observed in certain traits. No statistically significant differences were detected between breeds for withers height (137.5 ± 2.7 and 137.0 ± 4.3 cm; *p* = 0.519), body length (144.8 ± 7.7 and 142.3 ± 5.4 cm; *p* = 0.068), or forechest width (18.8 ± 1.5 and 19.3 ± 1.6 cm; *p* = 0.104). However, rump height was significantly higher in Holstein cattle (141.8 ± 2.8 cm) compared to Simental cattle (139.9 ± 5.2 cm; *p* = 0.021). No significant breed differences were observed for withers height, body length, or forechest width (*p* > 0.05). However, rump height was significantly greater in Holstein cattle compared to Simmental cattle (*p* = 0.021).

Table 2 summarizes the systematic differences (bias) between tape and camera measurements. In the Holstein group, significant differences were observed for withers height (−0.94 cm; *p* = 0.008) and forechest width (−0.46 cm; *p* = 0.03), whereas no significant difference was detected for rump height (*p* = 0.20). Body length showed borderline statistical significance (*p* = 0.050).

In the Simental group, camera measurements were significantly higher than tape measurements for all evaluated traits (withers height: −0.80 cm; body length: −1.08 cm; rump height: −1.26 cm; forechest width: −0.48 cm; all *p* < 0.01). Overall, the camera system demonstrated a consistent tendency to produce higher measurements in Simmental cattle.

Regression analysis revealed a statistically significant relationship between image-based and traditional measurements in Simmental cattle (*p* < 0.05), whereas no significant relationship was observed in Holstein cattle (*p* > 0.05). Differences in regression significance between breeds may be associated with breed-specific morphological variability and body conformation differences, which can affect the accuracy of image-based measurements. Table 3 presents the predictive power of camera measurements for estimating tape-measured values. In the Holstein group, the highest coefficient of determination was observed for body length (R^2^ = 0.961), indicating a very strong linear relationship. Moderate explanatory power was detected for withers height (R^2^ = 0.517) and rump height (R^2^ = 0.430), while forechest width showed relatively low predictive accuracy (R^2^ = 0.270).

**Figure 2:**
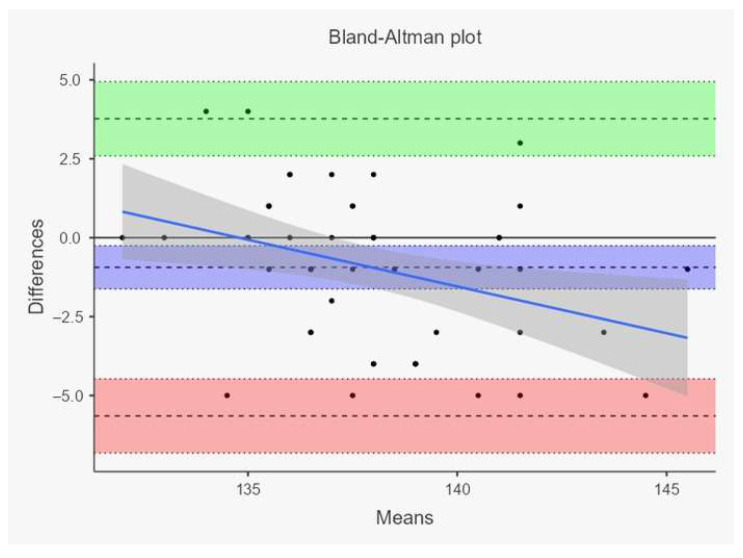
Bland–Altman plots showing the agreement between traditional tape measurements and smartphone-camera-based measurements in Holstein cattle for withers height, body length, rump height, and forechest width.

**Figure 3:**
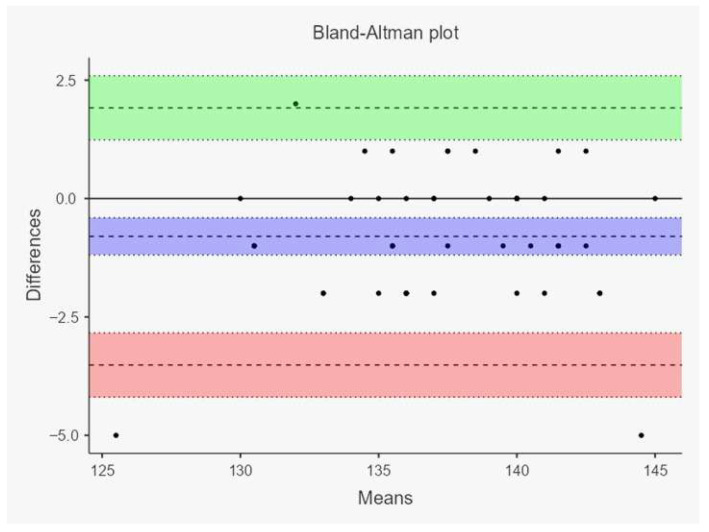
Bland–Altman plots illustrating the agreement between traditional tape measurements and smartphone-camera-based measurements in Simmental cattle for withers height, body length, rump height, and forechest width.

In the Simental group, very high coefficients of determination were observed for withers height (R^2^ = 0.895) and body length (R^2^ = 0.907), while rump height (R^2^ = 0.740) and forechest width (R^2^ = 0.564) showed moderate to high correlations. These high coefficients of determination indicate a strong linear relationship between image-based and traditional measurements, particularly for body length and withers height. However, a high R^2^ value reflects correlation rather than true agreement between methods. Bland–Altman analysis demonstrated the presence of systematic bias and variability between measurement techniques, indicating that the two methods should not be considered fully interchangeable for all morphometric traits. The observed differences were generally small in magnitude but should nevertheless be taken into account when interpreting image-based measurements under field conditions. Simple linear regression analyses were also performed to quantify these relationships; for example, withers height: Y = 0.12 + 0.98X, *p* < 0.001; body length: Y = 1.05 + 0.95X, *p* < 0.001, demonstrating that all slopes were highly significant. However, it should be emphasized that a high coefficient of determination (R^2^) reflects the strength of the linear relationship rather than true agreement between methods. Bland–Altman analysis revealed the presence of systematic bias, indicating that the two measurement techniques are not perfectly interchangeable despite their strong correlation (Figure 2 and Figure 3).

In each plot, the horizontal solid red line represents the mean bias (average difference between tape and camera measurements), while the upper and lower dashed blue lines indicate the 95% limits of agreement (mean bias ± 1.96 SD). The scattered black points correspond to individual animals and represent the relationship between the average of the two measurement methods (x-axis) and the difference between methods (y-axis). Values below zero indicate that smartphone-based measurements tended to produce slightly higher values than tape measurements. Narrower limits of agreement observed particularly for body length suggest stronger consistency between methods, whereas wider dispersion in rump height and forechest width indicates greater variability and lower agreement for anatomically more complex regions.

The solid red line indicates the mean bias between the two measurement techniques, whereas the dashed blue lines represent the 95% limits of agreement. Each black dot represents an individual animal measurement, plotted according to the mean value of the two methods and their corresponding difference. Negative bias values demonstrate that smartphone-camera-based measurements generally yielded slightly higher values compared to tape measurements. The relatively narrow distribution of points around the mean bias for withers height and body length indicates stronger agreement, while broader dispersion patterns for rump height and forechest width reflect increased measurement variability associated with posture, body conformation, and anatomical landmark identification.

## 4. Discussion

The body size characteristics of cows are one of the primary quality evaluation criteria to evaluate the growth response, which is influenced by nutrient supply and health anomalies. The measurement of cow body size was done manually, which has high labor costs, low accuracy, and easily causes stress in dairy cows. Therefore, it is of great significance to measure the body size of cows accurately and automatically using non-contact-based methods [26].

The consistency of body measurement values obtained in Simmental cattle with results reported in the literature supports the validity of the method used. Single-view approaches evaluated within the scope of non-contact measurement techniques allow for the practical determination of animals’ morphological characteristics. However, due to the fixed camera position and limited viewing angle, they make it difficult to accurately obtain multidimensional measurements such as chest circumference and abdominal circumference [27,28,29,30]. In this context, the use of a fixed camera is considered a methodological limitation in our study.

The complex structure of the farm environment, animal mobility, and physical obstacles that restrict the field of view can cause data gaps in point clouds obtained using multi-view approaches. These gaps have been reported to negatively affect measurement accuracy and visualization quality. Therefore, a decrease in success rate was anticipated, and multi-camera systems were not included in the scope of the research. However, it has also been noted that collecting data from different angles using multi-camera systems could improve measurement accuracy [31]. In this study, we attempted to prove the accuracy of the measurement by performing four repetitions for each measurement.

Studies demonstrating the usability of mobile devices in determining animal morphometry show that smartphones can serve as an alternative measurement tool. For example, one study reported that cow images were recorded using a Huawei P20 smartphone and that the distance between the device and the animal was approximately 1 m. The use of an iPhone 16 (smartphone) device in our study and the provision of similar distance conditions support methodological comparability.

It has previously been reported that technological measurement tools tend to produce relatively higher values compared to classical methods [13,18,31]. A similar trend was observed in our study, and the systematic tendency of camera-based measurements to produce slightly higher values than traditional measurements may be explained by several methodological and biological factors. Hair coat thickness may enlarge visible body contours in digital images, while slight postural deviations and respiratory movements can alter the apparent position of anatomical landmarks during image capture. In addition, the manual identification of reference points on two-dimensional images may introduce small operator-dependent deviations. Breed-specific body conformation differences, particularly in muscling and thoracic structure, may also contribute to variation in measurement accuracy between Holstein and Simmental cattle.

The magnitude of systematic bias observed in the present study generally ranged between approximately 0.5 and 1.3 cm, depending on breed and morphometric trait. Although these differences are relatively small from a practical perspective, they indicate that smartphone-based measurements may consistently overestimate certain body dimensions compared with direct tape measurements. Therefore, image-based measurements should be interpreted with consideration of potential method-dependent bias, particularly when precise morphometric evaluation is required.

Ensuring that animals remain motionless during the data collection process is critical for measurement accuracy, and it is recommended that the measurement time be approximately one minute per animal. However, traditional measurement methods have significant disadvantages, including risks to operator safety and animal welfare, as well as the inevitability of measurement errors [13,32]. In our study, camera-based measurement is completed in a shorter time and does not require contact, demonstrating the operational advantages of this method. The strong correlations observed between traditional and image-based measurements demonstrate that the two methods follow similar linear trends. Nevertheless, correlation alone does not indicate perfect agreement or interchangeability between methods. Bland–Altman analysis revealed systematic differences between measurement techniques, with camera-based measurements tending to produce slightly higher values in several traits. These discrepancies may be associated with factors such as hair coat thickness, body posture, respiratory movement, anatomical landmark identification, and breed-specific morphological characteristics [13,18,31]. It is anticipated that future studies with optimized camera positioning and better control of animal movements may further reduce the differences between the methods.

The fact that similar results were obtained in terms of measured regions in Holstein and Simmental cattle breeds demonstrates the applicability of image-based measurement techniques for different genotypes [13,20,33]. However, the existence of studies that collect data from different angles using multi-camera systems highlights the methodological limitation of our study, in which measurements were taken only from the left side [30]. Nevertheless, the evaluation of 100 animals is one of the strengths of the study in terms of sample size. In particular, animals with more uniform body structure and stable posture may yield more consistent results in image-based measurements, which could explain the stronger relationships observed in Simmental cattle.

An important limitation of the present study is that all image-based measurements were obtained from a single lateral view. Although this approach increases practicality under field conditions, two-dimensional imaging may not fully capture anatomically complex or depth-dependent body regions. Consequently, traits such as forechest width and rump height may be more sensitive to posture, viewing angle, and landmark identification errors. Multi-view imaging systems and three-dimensional reconstruction approaches may provide more accurate morphometric assessment in future studies.

When compared to studies reporting specific ranges for withers height, rump height, and body length in Holstein cows, measurements in our study were generally lower but showed a similar distribution [34]. These differences are thought to stem from variability in care, feeding, and breeding conditions.

Numerous studies have indicated that body measurements are related to production parameters such as growth, development, and live weight estimation [34,35,36,37]. However, it has been stated that measuring live weight with scales causes stress in animals and increases labor requirements [9]. Stress is known to negatively affect animal productivity and welfare [38,39]. While this study emphasizes the importance of body measurements in terms of production parameters, no analysis was performed in this direction, as the scope of the research did not directly involve yield estimation. The lack of focus on more parameters was a weakness of the study.

With advancements in technology, many measurements can be taken on animals using high-resolution instruments such as ultrasound and drones. The results of our study can be integrated with the results of these studies [40,41,42].

Recent studies have highlighted that animal measurements performed using technological devices yield similar results to traditional methods and are easier and safer [13,40,41,42].

A study conducted to improve the automated BCS classification by expanding the inclusion of body condition–related features extracted from multiple body regions using 3-D cameras [29]. This study considered BCS an ordinal variable and included all extracted features from multiple 3-D cameras in a BCS classification model. We increased the sensitivity of BCS classification compared with that reported for current machine vision–based body condition scoring methods. While repeated measurements yielded accurate results in our study, the findings of a previous study may be more relevant for modern farms. The ease of application of our study may be advantageous in field conditions.

Animal welfare is guaranteed, and risk is decreased by replacing human measurements with technological tools like mobile phones and cameras. One of the primary selection factors in cow breeding, along with performance and health, is conformation [43].

## 5. Conclusions

This study compared traditional tape measurements and smartphone-camera-based morphometric measurements in Holstein and Simmental cattle under field conditions. Strong linear relationships were identified between methods for several morphometric traits, particularly withers height and body length. However, Bland–Altman analyses demonstrated the presence of systematic differences between methods, indicating that high correlation does not necessarily imply complete interchangeability. Camera-based measurements generally produced slightly higher values than tape measurements, particularly in anatomically complex regions. Despite these differences, smartphone-based measurements may provide a practical, rapid, and contactless alternative for routine morphometric evaluation in cattle production systems. Nevertheless, measurements were obtained only from a single lateral view, which represents an important methodological limitation. Future studies integrating multi-view imaging systems, automated landmark detection, and three-dimensional analysis may improve measurement precision and reduce method-dependent variability.

## Figures and Tables

**Table 1:** Comparison of tape-measured morphometric traits between breeds.

Trait	Holstein (Mean ± SD)	Simental (Mean ± SD)	*p*-Value
Withers Height (cm)	137.5 ± 2.7	137.0 ± 4.3	0.519
Body Length (cm)	144.8 ± 7.7	142.3 ± 5.4	0.068
Rump Height (cm)	141.8 ± 2.8	139.9 ± 5.2	0.021
Forechest Width (cm)	18.8 ± 1.5	19.3 ± 1.6	0.104

**Table 2:** Bias analysis between tape and camera measurements.

Breed	Trait	Bias (Tape—Camera)	*p*-Value
Holstein	Withers Height	−0.94	0.008
Holstein	Body Length	−0.48	0.050
Holstein	Rump Height	−0.46	0.20
Holstein	Forechest Width	−0.46	0.03
Simental	Withers Height	−0.80	<0.001
Simental	Body Length	−1.08	<0.001
Simental	Rump Height	−1.26	0.002
Simental	Forechest Width	−0.48	0.004

**Table 3:** Linear regression results (camera predicting tape measurements).

Breed	Trait	Regression Equation	Intercept	Slope	R^2^	*p*-Value
Holstein	Withers height	Y = 60.464 + 0.556X	60.464	0.556	0.517	<0.001
Holstein	Body length	Y = −17.360 + 1.120X	−17.360	1.120	0.961	<0.001
Holstein	Rump height	Y = 63.304 + 0.552X	63.304	0.552	0.430	<0.001
Holstein	Forechest width	Y = 8.793 + 0.521X	8.793	0.521	0.270	<0.001
Simmental	Withers height	Y = 2.689 + 0.975X	2.689	0.975	0.895	<0.001
Simmental	Body length	Y = 11.278 + 0.914X	11.278	0.914	0.907	<0.001
Simmental	Rump height	Y = 28.747 + 0.803X	28.747	0.803	0.740	<0.001
Simmental	Forechest width	Y = 4.705 + 0.738X	4.705	0.738	0.564	<0.001

## Data Availability

The original contributions presented in this study are included in the article. Further inquiries can be directed to the corresponding author.

## References

[1] Kelgökmen İ., Ünal N. (2015). Anadolu mandıralarında bazı morfometrik ölçümler. Lalahan Hay. Araşt. Enst. Derg..

[2] Berry D.P., Buckley F., Dillon P., Evans R.D., Rath M., Veerkamp R.F. (2003). Genetic relationships among body condition score, body weight, milk yield, and fertility in dairy cows. J. Dairy Sci..

[3] Du A., Guo H., Lu J., Su Y., Ma Q., Ruchay A., Marinello F., Pezzuolo A. (2022). Automatic livestock body measurement based on keypoint detection with multiple depth cameras. Comput. Electron. Agric..

[4] Hu H., Xu Z., Han L., Qiao Z., Wang Y., Jia Y., Mu T., Ma Y. (2025). Genetic Analysis of Stayability and its Relationships with Production, Conformation, Fertility and Health Traits in Holstein Cattle. Vet. Sci..

[5] Koç A., Akman N. (2007). Body Measurements of Holstein-Friesian Bulls at Different Periods and Live Weight Prediction From Body Measurements. J. Adnan Menderes Univ. Agric. Fac..

[6] Heinrichs A.J., Erb H.N., Rogers G.W., Cooper J.B., Jones C.M. (2007). Variability in Holstein heifer heart-girth measurements and comparison of prediction equations for live weight. Prev. Vet. Med..

[7] Sharma B., Koundal D. (2018). Cattle health monitoring system using wireless sensor network: A survey from innovation perspective. IET Wirel. Sens. Syst..

[8] Arshad J., Siddiqui T.A., Sheikh M.I., Waseem M.S., Nawaz M.A.B., Eldin E.T., Rehman A.U. (2023). Deployment of an intelligent and secure cattle health monitoring system. Egypt. Inform. J..

[9] Coşkun G., Şahin Ö., Özkan İ.A., Aytekin İ. (2022). Comparison of Data Mining Algorithms used in Predictive of Live Weight from Body Measurements in Holstein Cattle at Different Growth and Development Periods. Agric. Eng..

[10] Yavuz S., Kaygısız A. (2015). The Relationship Between Some Body and Udder Measurements with Somatic Cell Count in Holstein Cows. KSU J. Nat. Sci..

[11] Gürcan E.K., Tuna Y.T., Soysal M.İ. (2011). The Morphometric Characterization of Anatolian Water Buffalo According to Body Measurements. J. Tekirdag Agric. Fac..

[12] Koçyiğit R., Yanar M., Aydın R., Diler A., Güler O. (2017). A Study on Milking Management Applied in Cattle Enterprises in Narman County of Erzurum Province. Alinteri J. Agric. Sci..

[13] Ünal N., Paksoy Y., Güngör Ö.F. (2025). Agreement of the morphometric dimensions determined by traditional and image analysis methods for Arabian and Thoroughbred horses. Arch. Anim. Breed..

[14] Şahin O. (2021). The Structure of Beef Production and Determination of Some Body Measurements in Fattened Cattle in Bolu Province. KSU J. Nat. Sci..

[15] Önal A.R., Dama E., Tuna Y.T. (2021). Relationship Between Production Characteristics and Proportion of Body Measurements of Holstein Cows. KSU J. Agric. Nat..

[16] Giloyan G.A., Kasumyan N.A. (2023). Technology of raising breeding replacement stock of the Fleckvieh breed. BIO Web Conf..

[17] Koçak S., Özbeyaz C. (2005). Production Traits of Kilis, Simmental x Kilis F1, B1 and F1xB1 Genotypes. J. Lalahan Anim. Husb. Res. Inst..

[18] Freitag G.P., de Lima L.G.F., Jacomini J.A., Kozicki L.E., Ribeiro L.B. (2021). An Accurate Image Analysis Method for Estimating Body Measurements in Horses. J. Equine Vet. Sci..

[19] Mariz T., Santos W., Mota L., Martins R., Lima C., Escodro P., Lima Junior D., Oliveira L., Sousa M., Ribeiro J. (2015). Evaluation morphostructural measures in horses of the Quarter horse Breed using image analysis. Acta Vet. Bras..

[20] White J.M., Mrllor D.J., Duz M., Lischer C.J., Voute L.C. (2008). Diagnostic accuracy of digital photography and image analysis for the measurement of foot conformation in the horse. Equine Vet. J..

[21] Paukovic D., Ilic T., Maletic M., Jovanovic N.M., Nedic S., Mirilovic M., Nenadovic K. (2025). Welfare on Dairy Cows in Different Housing Systems: Emphasis on Digestive Parasitological Infections. Vet. Sci..

[22] Ünal N., Keçelï H.H., Gündoğar U.C., Yakan A., Özkan H., Onbaşilar E., Yüceer Özkul B., Özbeyaz C. (2025). Foremilk fat content significantly related to expressions of microRNAs, fatty acid composition, and milk quality parameters of milk in Holstein cows. J. Food Compos. Anal..

[23] Wangchuk K., Wangdi J., Mindu M. (2018). Comparison and reliability of techniques to estimate live cattle body weight. J. Appl. Anim. Res..

[24] (2023). IPhone: Measure Dimensions with iPhone. https://support.apple.com/guide/iphone/measure-dimensions-iphd8ac2cfea/ios#:~:text=Use.

[25] Shoimah U.S., Dakhlan A., Sulastri, Hamdani M.D.I. (2021). Use of body measurements to predict live body weight of Simmental bull in Lembang Artificial Insemination Center, West Java. IOP Conf. Ser. Earth Environ. Sci..

[26] Yang G., Xu X., Song L., Zhang Q., Duan Y., Song H. (2022). Automated measurement of dairy cows body size via 3D point cloud data analysis. Comput. Electron. Agric..

[27] Zhang X.Y., Liu G., Jing L., Si Y.S., Ren X.H., Ma L. (2019). Automatic extraction method of cow’s back body measuring point based on simplification point cloud. Trans. Chin. Soc. Agric. Mach..

[28] Weber A.M., de Lima Weber F., da Silva Oliveira A., Astolfi G., Menezes G.V., de Andrade Porto J.V., Pistori H. (2020). Cattle weight estimation using active contour models and regression trees Bagging. Comput. Electron. Agric..

[29] Song X., Bokkers E.A.M., Van Mourik S., Koerkamp P.G., Van Der Tol P.P.J. (2019). Automated body condition scoring of dairy cows using 3-dimensional feature extraction from multiple body regions. J. Dairy Sci..

[30] Shi C., Zhang J., Teng G. (2019). Mobile measuring system based on LabVIEW for pig body components estimation in a large-scale farm. Comput. Electron. Agric..

[31] Zheng Z., Gao J.B., Weng Z. (2024). Measurement of body size parameters and body weight prediction in beef cattle based on image analysis. J. Intell. Fuzzy Syst..

[32] dos Santos M.R., Freiberger G., Bottin F., Chiocca M., Zampar A., de Córdova Cucco D. (2017). Evaluation of methodologies for equine biometry. Livest. Sci..

[33] van der Vlugt-Meijer R.H., Meij B.P., Voorhout G. (2006). Intraobserver and interobserver agreement, reproducibility, and accuracy of computed tomographic measurements of pituitary gland dimensions in healthy dogs. Am. J. Vet. Res..

[34] Tasdemir Ş., Urkmez A., Inal Ş. (2011). Determination of body measurements on the Holstein cows using digital image analysis and estimation of live weight with regression analysis. Comput. Electron. Agric..

[35] Rao Dharma M.V., Seshaiah C.V., Rao Jagadeeswara S., Vinoo R., Kumar Srinivas D. (2020). Relationship between Morphometric and Milk Production Characters in Ongole Cattle. Indian J. Anim. Res..

[36] Bardakcioglu H.E., Sekkin S., Oral Toplu H.D. (2011). Relationship between some teat and body measurements of Holstein cows and sub-clinical mastitis and milk yield. J. Anim. Vet. Adv..

[37] Bhuiyan M.M., Islam M.R., Ali M.L., Hossain M.K., Kadri M.A., Lucky N.S., Das B.R. (2004). Importance of mammary system conformation traits in selecting dairy cows on milk yield in Bangladesh. J. Biol. Sci..

[38] Babiciu D.E., Blaga Petrean A., Daina S., Neagu D.M., Lazar E.A., Popescu S. (2026). Milk Biomarkers and Herd Welfare Status in Dairy Cattle: A Machine Learning Approach. Vet. Sci..

[39] Yao L., Liu J., Hong W., Kong F., Fan Z., Lei L., Li X. (2025). SideCow-VSS: A Video Semantic Segmentation Dataset and Benchmark for Intelligent Monitoring of Dairy Cows Health in Smart Ranch Environments. Vet. Sci..

[40] Los S., Mücher C.A., Kramer H., Franke G.J., Kamphuis C. (2023). Estimating body dimensions and weight of cattle on pasture with 3D models from UAV imagery. Smart Agric. Technol..

[41] Wang Y., Mücher S., Wang W., Kooistra L. (2024). Automated retrieval of cattle body measurements from unmanned aerial vehicle-based LiDAR point clouds. Comput. Electron. Agric..

[42] AlZubi A.A. (2023). Application of Machine Learning in Drone Technology for Tracking Cattle Movement. Indian J. Anim. Res..

[43] Ferreira Padilha F.G., de Andrade A.M., Monteiro Fonseca A.B., de Godói F.N., de Almeida F.Q., Reis Ferreira A.M. (2017). Morphometric measurements and animal-performance indices in a study of racial forms of Brazilian Sport Horses undergoing training for eventing. R. Bras. Zootec..

